# Social patterning of subjective life expectancy in late midlife

**DOI:** 10.1093/geroni/igaf122.900

**Published:** 2025-12-31

**Authors:** Anu Siren

**Affiliations:** Tampere University, Tampere, Pirkanmaa, Finland

## Abstract

Subjective life expectancy (SLE), an individual’s personal estimate of their lifespan, impacts various aspects of life, including retirement planning and health behaviors. Due to increasing longevity and changing institutions such as pension systems and statutory insurances, individuals are increasingly compelled to adapt to the prospect of longevity and weigh the risks of aging. However, the ability to envision a long life is socially stratified and influenced by factors such as gender, socioeconomic status, and health. Notwithstanding, relatively little is known about the social patterning of SLE. Using various measures of lifestyle such as social participation, cultural consumption, and health lifestyle, this study investigates how SLE is determined in late midlife and how this determination is socially patterned. The data comes from a nationally representative survey (n = 3327) in Finland among people born in 1960, 1965, and 1970. We employ principal components analysis and logistic regression and find that, in addition to female gender, good health, and high income, certain lifestyles and cultural consumption patterns independently predict higher SLE. The results contribute to the literature on the social stratification of SLE, suggesting that while more socially varied populations live longer, the prospect of a long life seems imaginable only for rather select subgroups of people. Increasing longevity prompts societal negotiation of risks related to long life courses. Nevertheless, it is important to acknowledge that a great variation exists at the individual level in people’s ability and willingness to commit to the long futures and to manage the risks of aging.

